# The effect of androgen excess on maternal metabolism, placental function and fetal growth in obese dams

**DOI:** 10.1038/s41598-017-08559-w

**Published:** 2017-08-14

**Authors:** Romina Fornes, Manuel Maliqueo, Min Hu, Laila Hadi, Juan M. Jimenez-Andrade, Kerstin Ebefors, Jenny Nyström, Fernand Labrie, Thomas Jansson, Anna Benrick, Elisabet Stener-Victorin

**Affiliations:** 1 Department of Physiology and Pharmacology, Karolinska Institutet, 171 77 Stockholm, Sweden; 2 Endocrinology and Metabolism Laboratory, Department of Medicine, West Division, University of Chile, Santiago, Chile; 3 Department of Physiology, Institute of Neuroscience and Physiology, Sahlgrenska Academy, University of Gothenburg, Gothenburg, Sweden; 4 Unidad Académica Multidisciplinaria Reynosa Aztlán, Universidad Autónoma de Tamaulipas, Reynosa, Tamaulipas Mexico; 5 Laval University Research Center in Molecular Endocrinology, Oncology and Human Genomics, CHUL Research Center, Quebec, G1V 4G2 Canada; 6 Department of Obstetrics & Gynecology, Division of Reproductive Sciences, University Colorado Anschutz Medical Campus, Aurora, Colorado 80045 USA; 7 School of Health and Education, University of Skövde, 54128 Skovde, Sweden

## Abstract

Pregnant women with polycystic ovary syndrome (PCOS) are often overweight or obese. To study the effects of maternal androgen excess in obese dams on metabolism, placental function and fetal growth, female C57Bl6J mice were fed a control (CD) or a high fat/high sucrose (HF/HS) diet for 4–10 weeks, and then mated. On gestational day (GD) 15.5–17.5, dams were injected with dihydrotestosterone (CD-DHT, HF/HS-DHT) or a vehicle (CD-Veh, HF/HS-Veh). HF/HS dams had higher fat content, both before mating and on GD18.5, with no difference in glucose homeostasis, whereas the insulin sensitivity was higher in DHT-exposed dams. Compared to the CD groups, the livers from HF/HS dams weighed more on GD18.5, the triglyceride content was higher, and there was a dysregulation of liver enzymes related to lipogenesis and higher mRNA expression of *Fitm1*. Fetuses from HF/HS-Veh dams had lower liver triglyceride content and mRNA expression of *Srebf1c*. Maternal DHT exposure, regardless of diet, decreased fetal liver *Pparg* mRNA expression and increased placental androgen receptor protein expression. Maternal diet-induced obesity, together with androgen excess, affects maternal and fetal liver function as demonstrated by increased triglyceride content and dysfunctional expression of enzymes and transcription factors involved in *de novo* lipogenesis and fat storage.

## Introduction

Androgen excess in females is associated with insulin resistance, β-cell failure, ovarian dysfunction and infertility^1^. Moreover, around 28 to 64% of women with polycystic ovary syndrome (PCOS) are obese which increases the risk of poor pregnancy outcomes^2, 3^. Furthermore, when women with PCOS are pregnant, hyperandrogenemia is linked to adverse obstetric outcomes, including gestational diabetes mellitus (GDM), preeclampsia and low *or* high birth weight^4, 5^. It has been suggested that an impairment in the decidual trophoblast invasion^6^, and/or alterations of the microstructure of the placenta^7^, may be involved in the increased risk of pregnancy complications in women with PCOS. Exposure to an adverse intrauterine environment in pregnancies complicated by PCOS may program the fetus for long-term health consequences, including reproductive dysfunction as well as behavioral and metabolic abnormalities^8–11^.

It has been proposed that PCOS originates during fetal development and that this might be due, in part, to maternal androgen excess^9–12^. Maternal obesity, either established prior to pregnancy or due to an excessive weight gain during pregnancy, increases the risk of GDM and gestational hypertension and is associated with adverse obstetric outcomes including preterm birth and cesarean delivery^13^. Thus, maternal obesity is another factor causing an unfavorable intrauterine environment and it is associated with increased birth weight in the offspring and an augmented risk of being overweight at 4 years^14, 15^ and at 21 years of age^16^.

A large number of models of maternal obesity have been developed in various species. In some of these models maternal obesity is associated with altered placental function^17^, fetal overgrowth^18^, offspring hypertension, glucose intolerance^19^, stress^20^ and an altered methylation profile in oocytes and offspring liver, predisposing to the development of metabolic failures^21^.

Maternal (prenatal) androgen exposure is a commonly used animal model of PCOS that has been developed in rodents, sheep and non-human primates^8–12, 22, 23^. All these models reflect the lean PCOS phenotype and fail to reproduce the combination of maternal androgen excess *and* maternal obesity, and might not be relevant to overweight/obese pregnant women with PCOS. However, in the nonhuman primate PNA model, testosterone propionate have shown to increase the maternal body weight gain together with an altered maternal glucoregulation and higher body weight of the offspring at 8 weeks of postnatal life^24^. In fact, high levels of androgens during pregnancy have been related to metabolic^25^ and cardiovascular^26^ derangements. In animal models, we and others have also shown that prenatally androgenized (PNA) female rat offspring are born small for gestational age (SGA), but normal fetal weight has also been reported^9–11, 22, 27, 28^. However, in pregnant women with PCOS, especially those that are obese, both SGA and large for gestational age (LGA) occur^29, 30^. The PNA rat offspring are not obese but exhibit metabolic disturbances and dysfunction of adipose tissue, as reflected by altered adipocyte size, high circulating triglycerides (TG), non-alcoholic fatty liver and reproductive dysfunction^22^. Thus, there is a need for an animal model of both androgen excess and maternal obesity that is more reflective of human PCOS pregnancies complicated by obesity.

In the current study, we aimed to combine the PNA mouse model with a model of maternal obesity induced by a high fat/high sugar Western style diet that has been shown to result in fetal overgrowth^18, 31^ and to investigate maternal metabolism, placental function and fetal growth.

## Results

### Maternal Variables

**Figure 1:**
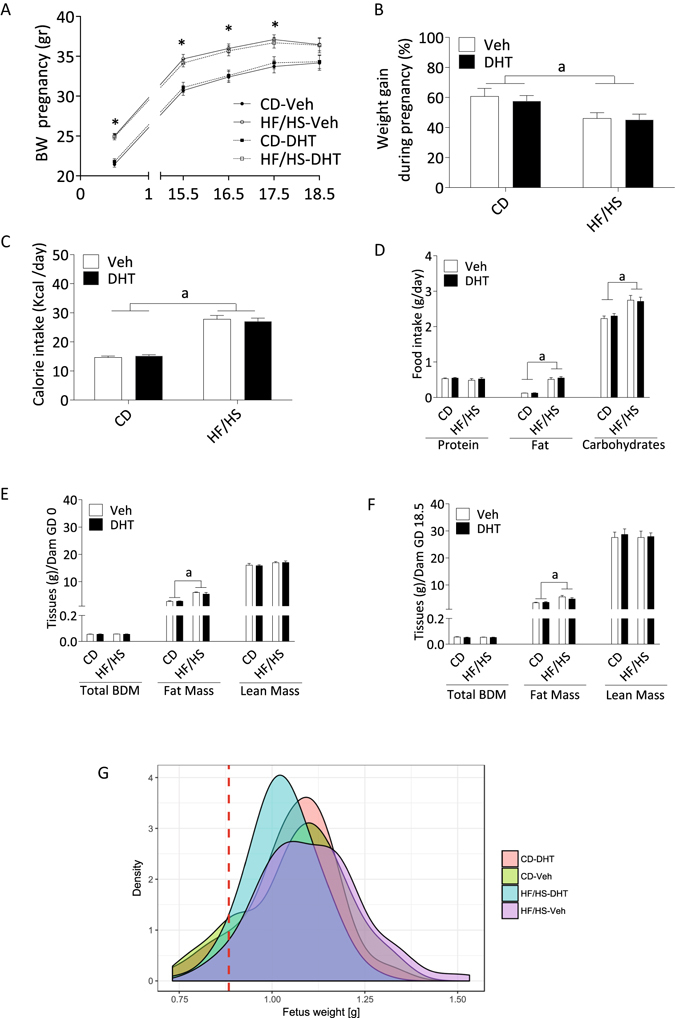
Maternal body weight development, calorie intake and body composition (**A**) Body weight gain during pregnancy; (**B**) Percentage of weight gain during pregnancy (CD-Veh, n = 12–13; HF/HS-Veh, n = 12–13; CD-DHT, n = 11–12; HF/HS-DHT, n = 14). (**C**) Caloric intake (Kcal/day) (CD-Veh, n = 12–13; HF/HS-Veh, n = 12–13; CD-DHT, n = 11–12; HF/HS-DHT, n = 14). (**D**) Food Intake (g/day) (**E**) DEXA the day before mating (GD0) and (**F**) DEXA GD18.5 (CD-Veh, n = 5; HF/HS-Veh, n = 5; CD-DHT, n = 4; HF/HS-DHT, n = 4). (**G**) Distribution of fetal weight across the groups. Values are means ± SEM. *P < 0.05 HF/HS *vs* CD-Veh and HF/HS-DHT vs CD-Veh or CD-DHT, a = Main effect of diet *p* < 0.05.

The body weight development during pregnancy is shown in Fig. 1A. Dams in the HF/HS-Veh and HF/HS-DHT groups weighed more than CD and CD-DHT dams from GD0 until GD17.5 (p < 0.05), but did not differ in body weight on GD18.5 (Fig. 1A).

There was a main effect of diet on weight gain during pregnancy; dams fed HF/HS had a lower percentage of weight gain than those fed control chow [F(1,44) = 10.4. p < 0.05] (Fig. 1B). Calorie intake (Fig. 1C) and also food intake per day, i.e. the number of grams of the sucrose solution and pellets, was higher in dams fed HF/HS [F(1,48) = 150.4. p < 0.001] (Fig. 1D).

There was a significant main effect of diet on body composition measured by DEXA prior to mating and on GD 18.5 with higher fat mass in dams fed HF/HS both on GD0 [F(1,14) = 129.1, p < 0.001] (Fig. 1E) and on GD18.5 [F (1,14) = 19.44, p < 0.001] (Fig. 1F), with no effect of the DHT injection.

The litter size and placental weight were not affected by maternal diet or DHT injection (Table 1). Although not significant, there was a trend for lower fetal weight in DHT injected dams than in vehicle-injected dams [F (1,48) 3.16, p = 0.082] (Table 1). Posthoc analyses revealed that it was fetuses from HF/HS-DHT dams that weighed less than fetuses from HF/HS-Veh dams (p = 0.035) (Table 1). The body composition of fetuses, assessed by BCA, was not affected by maternal diet or DHT injection (Table 2). The distribution of fetal weight is displayed in Fig. 1G.

**Table 1:** Litter size, fetal and placental weight.

	CD-Veh (n = 13)	CD-DHT (n = 13)	HF/HS-Veh (n = 12)	HF/HS-DHT (n = 14)	Main Effect
Litter size	7.62 ± 0.72	7.75 ± 0.69	7.69 ± 0.64	8.21 ± 0.59	ns
Fetal Weight at GD 18.5	1.07 ± 0.04	1.06 ± 0.03	1.11 ± 0.02	1.03 ± 0.02	ns
Placenta Weight at GD 18.5	0.10 ± 0.00	0.10 ± 0.00	0.10 ± 0.00	0.10 ± 0.00	ns
Fetus/Placenta Weight	10.99 ± 0.47	11.02 ± 0.30	11.75 ± 0.46	10.86 ± 0.29	ns

GD: Gestational day; CD, control diet; HF/HS, high fat diet; Veh, vehicle; DHT, dihydrotestosterone. ﻿ns: no significance. Data are Mean values ± SEM and were analyzed by 2-way ANOVA to investigate interactions between maternal diet and DHT injection or main effect of either maternal diet or DHT. There was no interaction between maternal diet and the DHT injection in any variables studied.

**Table 2:** Fetal body composition was assessed by Body Composition Analyzer (BCA), Bruker minispec LF110.

	CD-Veh n = 4	CD-DHT n = 5	HF/HS-Veh n = 4	HF/HS-DHT n = 4	Main Effect
Body Mass (g)	1.07 ± 0.05	0.99 ± 0.12	1.12 ± 0.02	1.04 ± 0.03	ns
Lean Mass (g)	0.52 ± 0.11	0.66 ± 0.05	0.54 ± 0.09	0.53 ± 0.03	ns
Fat (g)	0.38 ± 0.06	0.32 ± 0.04	0.39 ± 0.05	0.34 ± 0.04	ns

CD, control diet; HF/HS, high fat diet; Veh, vehicle; DHT, dihydrotestosterone. ﻿ns: no significance. Data are Mean values ± SEM and were analyzed by 2-way ANOVA to investigate interactions between maternal diet and DHT injection or main effects of either maternal diet or DHT.

### Maternal circulating sex steroids and glucose metabolism

Maternal sex steroid levels measured by LC-MS/MS are presented in Table 3. There was a main effect of maternal diet on circulating progesterone levels [F(1,30) 5.77, p = 0.023] with higher concentrations in dams fed HF/HS and injected with DHT than in dams on control chow and injected with DHT (p < 0.05). There was a main effect of the DHT injection with higher circulating DHT concentrations in dams fed control chow and injected with DHT than in the vehicle controls [F(1,30) 9.97, p = 0.004], with no difference in dams fed HF/HS. There were no interaction effects or main effects of diet or DHT injection on circulating estradiol and testosterone.

**Table 3:** Sex steroids in the serum of dams on GD 18.5.

	CD-Veh n = 7–8	CD-DHT n = 8	HF/HS n = 8	HF/HS-DHT n=10	Main Effect	Posthoc test (p < 0.05)
Estradiol (pg/ml)	19.21 ± 1.87	16.68 ± 1.48	23.77 ± 4.47	17.26 ± 2.19	ns	ns
Testosterone (pg/ml)	117.58 ± 15.77	155.48 ± 32.39	108.84 ± 10.77	116.60 ± 11.13	ns	ns
Progesterone (ng/ml)	16.20 ± 5.89	11.99 ± 4.42	26.40 ± 11.70	36.57 ± 8.21	a: p = 0.023	HF/HS-DHT vs CD-DHT
DHT (pg/ml)	45.80 ± 14.58	1553.51 ± 783.59	52.40 ± 11.44	961.44 ± 173.67	b: p = 0.004	CD-DHT vs CD-Veh

CD, control; HF/HS, high fat/high sucrose diet; DHT, dihydrotestosterone. Data are Mean values ± SEM. a = Main effect of diet [F (1,30) 5.77, p = 0.023]. b = Main effect of injection [F (1.30) 9.97, p = 0.004] There was no interaction between diet and DHT injection for any of the variables.

**Figure 2:**
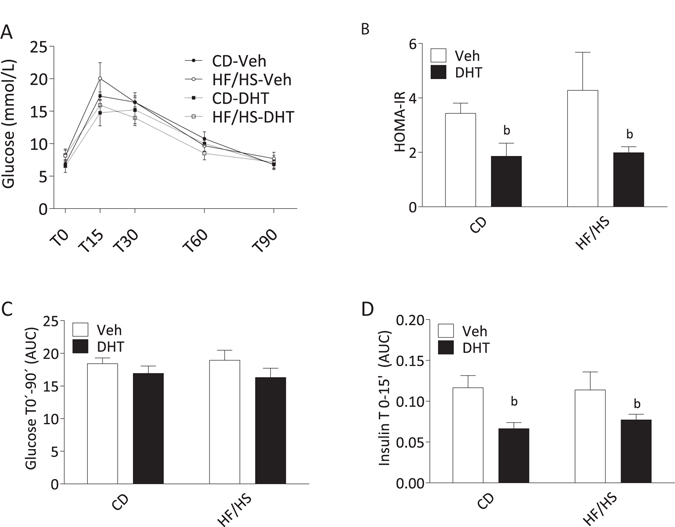
Glucose metabolism on GD 18.5. (**A**) Blood glucose levels during oral glucose tolerance test (OGTT) (n = 4–5 per group). (**B**) The Homeostasis Model Assessment (HOMA-IR). (**C**) Glucose area under the curve (AUC) T0-90 minutes and (**D**) Insulin AUC T0-15 minutes (n = 3–5 per group). Values are means ± SEM; b = Main effect of injection *p* < 0.05.

The glucose levels from the oral glucose tolerance test (OGTT), performed on GD18.5, are presented in Fig. 2A. There was a main effect of the DHT injection with lower Homeostasis Model Assessment Insulin Resistance (HOMA-IR) in DHT injected pregnant dams than in vehicle-injected dams [F(1,11) 5.40. p = 0.040] (Fig. 2B). There were no interaction or main effects of diet or DHT injection on the Area Under the Curve (AUC) of glucose T0-90′ (Fig. 2C). However, there was a main effect of the DHT injection with lower AUC of insulin T0-15′ [F(1,10) 8.06. p = 0.018] (Fig. 2D), specifically when dams fed control chow and injected with DHT were compared to vehicle controls (p = 0.043) (Fig. 2D).

### Placental steroidogenesis

**Figure 3:**
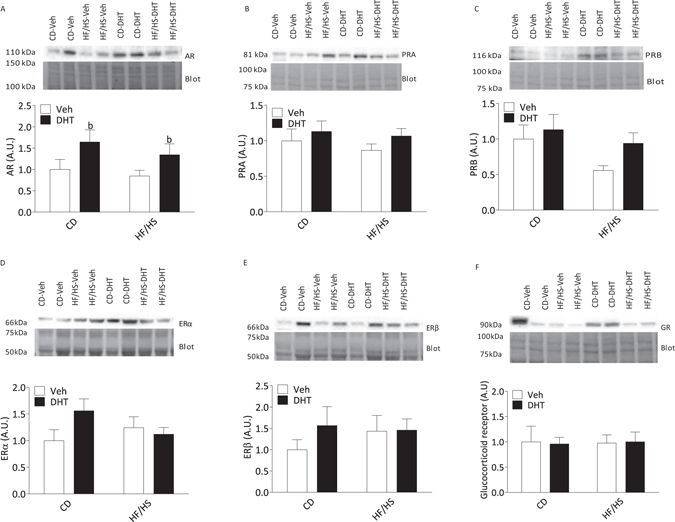
Protein expression of sex steroid receptors in placental homogenates on GD 18.5 (**A**) Androgen receptor (AR), (**B**) Progesterone receptor A (PRA). (**C**) Progesterone receptor B (PRB), (**D**) Estrogen receptor (ER) α, ERα. (**E**), Estrogen receptor β, ERβ. (**F**) Glucocorticoid Receptor (GR). (CD-Veh, n = 8; HF/HS-Veh, n = 8; CD-DHT, n = 8; HF/HS-DHT, n = 10). Protein expression was quantified by Western blot and normalized to total protein loaded in each line in the stain free blot. All blot images were cropped for publication, and the full images are is shown in Supplementary Fig. 5. Values are means ± SEM. a = Main effect of diet *p* < 0.05.

There was a main effect of the DHT injection with higher androgen receptor expression in DHT injected dams than in vehicle-injected dams [F(1,25) 5.90, p = 0.023] (Fig. 3A). There were no interaction or main effects of diet or DHT injection on the expression of estrogen receptors α and β, progesterone receptor A and B or glucocorticoid (Fig. 3B–F), nor in the expression of steroidogenic enzymes CYP11A1 and 17-βHSD2 (Supplementary Fig. 2A,B).

### Circulating adiponectin and local adiponectin receptor in placenta

Circulating HMW adiponectin was not affected by diet or DHT injection (Supplementary Fig. 3A). There were no interaction or main effects of diet or DHT injection on the placenta protein expression of adiponectin receptor 2 [F(1,29) 3.781, p = 0.059] (Supplementary Fig. 3B).

### Liver weight and triglyceride content

**Figure 4:**
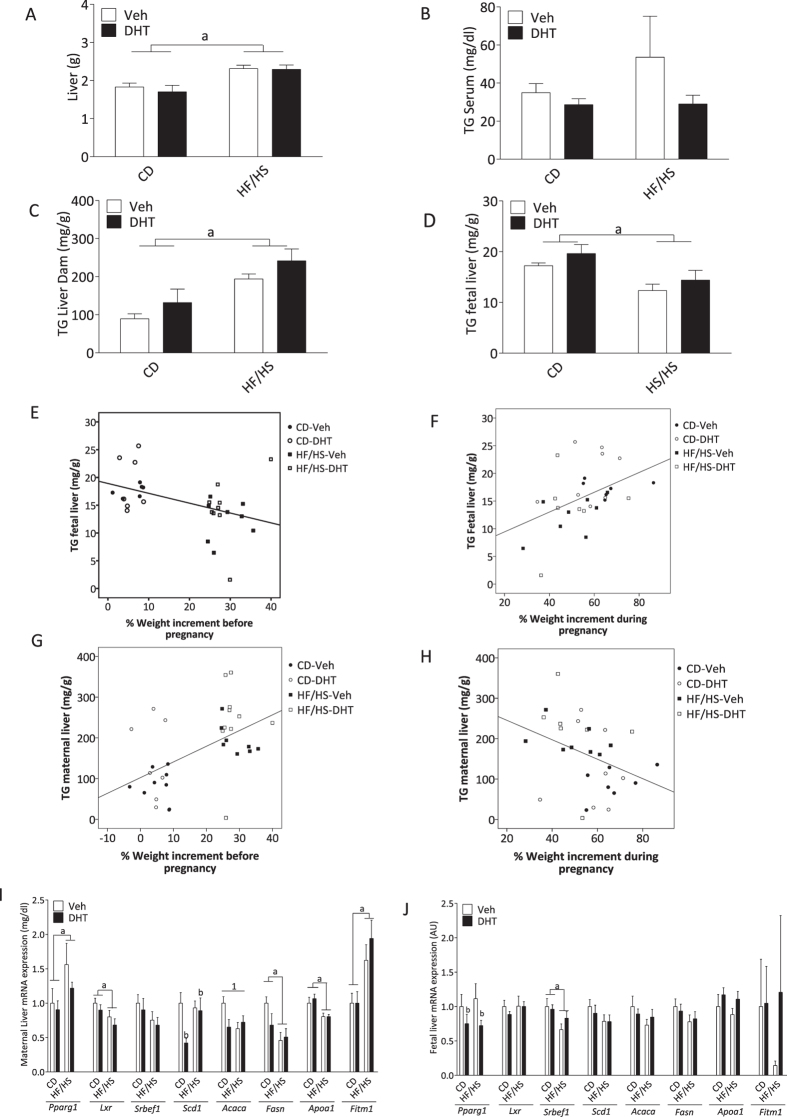
Liver weight, triglyceride content and gene expression of lipid metabolism markers in the maternal and fetal liver. (**A**) Weight of dams liver (CD-Veh, n = 8; HF/HS, n = 8; CD-DHT, n = 8; HF/HS-DHT, n = 10). (**B**) Circulating Triglycerides (TG) in serum and (**C**) TG content in dams liver, (CD-Veh, n = 8; HF/HS-Veh, n = 8; CD-DHT, n = 8; HF/HS-DHT, n = 9). (**D**) TG content in fetal liver (CD-Veh, n = 7; HF/HS, n = 8; CD-DHT, n = 8; HF/HS-DHT, n = 9). (**E**) Correlation analyses between TG content in fetal liver and maternal weight gain before pregnancy, (**F**) Correlation analyses between TG content in fetal liver and percentage of maternal weight gain during pregnancy, (**G**) Correlation analyses between TG content in maternal liver and percentage of maternal weight gain before pregnancy, (**H**) Correlation analyses between TG content in maternal liver and percentage of maternal weight gain during pregnancy, (**I**) Transcription factors, lipogenic enzymes, cholesterol and fat storage mediators in Maternal liver and (**J**) fetal liver (CD, n = 8; HF/HS, n = 8; CD-DHT, n = 7–8; HF/HS-DHT, n = 10). Values are means ± SEM. a = Main effect of diet, b = main effect of injection, 1 = Interaction between diet and injection.

There was a main effect of diet with higher liver weight in dams fed HF/HS than in dams fed CD [F(1,30) 52.40, p < 0.001] (Fig. 4A). Circulating TG levels were not affected by diet or injection. Although no interaction was found, there was a main effect of diet on maternal liver TG content with higher content in dams fed HF/HS [F(1,30) 16.50. p < 0.001], the posthoc test revealed that maternal liver TG content was higher in HF/HS-Veh and HF/HS-DHT than in the CD and CD-DHT groups, respectively (p < 0.05). Livers from DHT injected animals tended to have a higher content of DHT than their vehicle injected counterparts, although this effect was not statistically significant [F(1,30) 2.902. p = 0.09], (Fig. 4C). There was a main effect of diet on fetal liver TG content with lower content in fetuses from dams fed HF/HS than in fetuses from dams fed control chow [F(1,28) 6.92, p = 0.014] (Fig. 4D). Fetal TG content was inversely correlated with maternal weight gain before pregnancy (−0.452, p = 0.009, Fig. 4E), but positively correlated to pregnancy weight gain (0.446, p = 0.015, Fig. 4F). Contrary, the TG concentration in maternal liver correlated positively with pre-pregnancy weight gain (0.517, p = 0.002; Fig. 4G) and negatively with maternal weight gain in pregnancy (−0.365, p = 0.048, p = 0.015, Fig. 4H).

### Expression of genes related to triglyceride metabolism in the liver from dams and fetuses

In the maternal livers, there was a main effect of diet on mRNA expression of transcription factors; dams fed HF/HS had higher *Pparg* and lower *Lxr* than dams fed control chow [*Pparg*: F(1,30) 5.14, p = 0.031; *Lxr:* F(1,30) 6.12, p = 0.019] (Fig. 4I). There were both interaction effects and main effects of diet and DHT injection in the expression of enzymes related to the fatty acid synthesis. For the mRNA expression of *Scd1*, there was a main effect of the DHT injection [F(1,30) 4.69, p = 0.038] (Fig. 4I) with lower expression in DHT-injected than in vehicle-injected dams fed control chow (p < 0.01) and higher in the HF/HS-DHT than in the CD-DHT group (p = 0.023). For the mRNA expression of *Acaca*, there was an interaction between diet and DHT injection [F(1,30) 5.16, p = 0.030] (Fig. 4I). The posthoc analysis showed that *Acaca* had a lower expression in DHT-injected dams than in vehicle-injected dams fed control chow (p < 0.05), and lower expression in dams fed HF/HS than CD (p < 0.05). For enzymes related to fatty acid synthesis, there was a main effect of diet with lower mRNA expression of *Fasn* in dams fed HF/HS [F(1,30) 7.989. p < 0.01] (Fig. 4I) and of *Apoa1*, an important component of high density lipoprotein (HDL) [F(1,30) 14.66, p < 0.01] (Fig. 4I). Finally, there was a main effect of diet with higher mRNA expression of *Fitm1* in both HF/HS groups [F(1,30) 14.66, p < 0.01] (Fig. 4I).

There was a main effect of the DHT injection with lower mRNA expression of *Pparg* in fetal livers from DHT injected dams, than in fetal livers from vehicle-injected dams [F(1.29) 4.42, p < 0.05] (Fig. 4J), and a main effect of diet on *Srebfc* with lower mRNA expression in fetal livers from dams fed HF/HS [F(1.29) 6.44, p < 0.05] (Fig. 4J).

### Kidney

**Figure 5:**
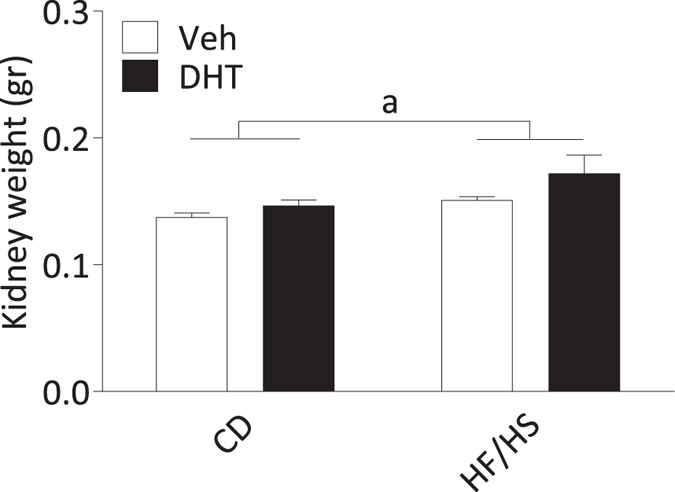
Kidney weight. The weight of the kidneys. a = Main effect of diet; *p* < 0.05 *vs* CD. Values are means ± SEM.

There was a main effect of diet with higher kidney weight in dams fed HF/HS than in controls [F(1,30) 4,250, p = 0.048] (Fig. 5A). The posthoc analysis demonstrated that kidneys in the HF/HS-DHT dams were heavier than the kidneys in the HF/HS dams injected with vehicle. Morphological evaluation of the renal tissues stained with PAS showed normally looking glomeruli with no difference between the four groups (data not shown).

## Discussion

Women with PCOS are often obese which increases the risk to deliver a LGA or SGA newborn. Many animal models of PCOS represent the lean phenotype of the syndrome with normal or growth restricted fetuses^22, 32^. The novelty of the present study is that we combine the well characterized PNA mouse model^9–11, 28^ with a maternal obesity model^18^. We demonstrate that diet-induced obesity results in enlarged maternal livers accompanied with a marked increase in maternal liver TG content, which was most pronounced in the DHT-treated animals. Moreover, HF/HS was associated with a decreased fetal liver TG content indicating a compensatory mechanism in the intrauterine life. As indicated by the HOMA-IR results, the HF/HS-diet induced obesity was not associated with changes in insulin sensitivity, whereas maternal androgen excess increased insulin sensitivity.

Studies in pregnant women with PCOS show that they gain more weight during pregnancy than controls, independent of body mass index (BMI)^30, 33^, and is more likely to give birth to either SGA^5^ or LGA^29^ babies. We here demonstrate that the total percentage of body weight gain in dams fed HF/HS was lower than in dams on control chow with no effect of the DHT injection during late pregnancy. The lower increase in body weight during late pregnancy is in line with another study using a similar HF/HS-induced obesity model with the same litter size^34^. Despite this, DEXA measurements show that dams fed HF/HS have higher fat mass than controls, both on GD0 and GD18.5, which is in line with a previous report with a similar HF/HS protocol^18^. In contrast to the results of Rosario *et al*.^18^, fetal weight was not higher in in fetuses from dams fed HF/HS. One plausible explanation could be the lower weight gain at the end of the pregnancy in dams fed HF/HS compared to dams on control chow.

As expected, the serum levels of DHT were higher in mice injected with DHT demonstrating that the dams were hyperandrogenized at the end of pregnancy. No previous studies using the maternal androgen excess model have measured DHT in maternal serum in mice^9–11, 28^. Since maternal androgens have the potential to affect the fetus and/or placental function directly^32^, it is important to investigate the circulating levels of this hormone. Thus, this is the first study to quantify maternal sex steroid concentrations in mice exposed to maternal androgen excess, with or without obesity. Circulating estradiol and testosterone was not affected by diet or the DHT injection, indicating that any changes observed in the DHT-exposed mice most likely are related to androgen receptor activation by DHT. However, an estrogen effect cannot completely be ruled out as DHT can be metabolized to 5α-androstane-3β,17β-diol (3β-diol) that could activate the estrogen receptor beta^35^.

Surprisingly, dams fed either HF/HS or CD and injected with DHT displayed a decrease in HOMA-IR. This indicates improved insulin sensitivity, rather than decreased, as hypothesized. Most likely, this reflects an acute effect of the DHT exposure. Our results are supported by a previous *in vitro* study of glucose-induced insulin secretion in cultured beta cells from normal rats^36^. Exposure for 72 hours to either DHT or testosterone reduced the insulin secretion, and the effect was reversed by flutamide, demonstrating the capacity of androgens to modulate insulin secretion via androgen receptors^36^.

As expected, the liver weight was increased in dams fed HF/HS. Although no effects of diet or DHT injection were found on circulating TG, the liver TG content was higher in dams fed HF/HS and in dams exposed to DHT. This finding is supported by a clinical study in hyperandrogenic women with PCOS, who exhibit higher liver fat content than women with PCOS without hyperandrogenism, independent of BMI and insulin resistance^37^. Moreover, the prevalence of nonalcoholic fatty liver disease in women with PCOS has been shown to be 25%^38^ often together with metabolic syndrome. All non-invasive indices of hepatic steatosis and hepatic fibrosis are higher than normal in women with PCOS, and even higher when the metabolic syndrome is present^39^. In addition, kidney weights were also significantly increased in the dams fed HF/HS and injected with DHT (Fig. 5A). Hyperandrogenic women with PCOS have previously been shown to have an increased risk for pre-term birth and preeclampsia^40^. However, in this study, we found no histological evidence of preeclampsia or other kidney injuries, which in this case may be due to a too short treatment time.

**Figure 6:**
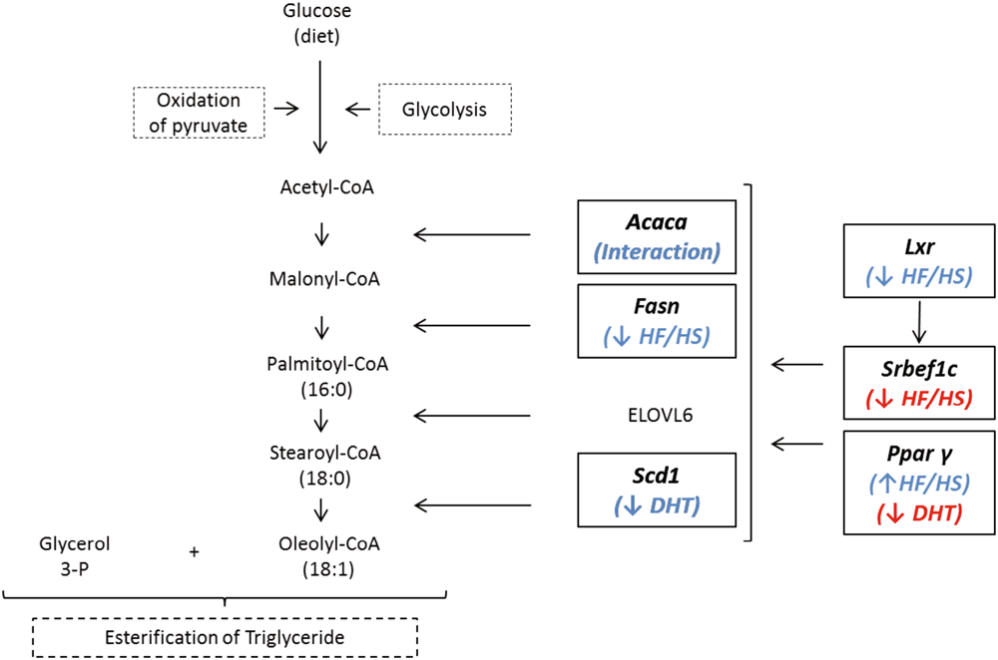
Simplified scheme of the enzymes and transcription factors involved in *de novo* lipogenesis pathway. Briefly, the enzymes Acetyl-CoA Carboxylase alpha (Acaca), Fatty acid synthase (Fasn), Elongation of very long chain fatty acids protein 6, (Elovl6), and Stearoyl-CoenzimeA desaturase, (Scd1) participate directly in the synthesis and elongation of fatty acids starting from Acetyl-CoA. Liver X receptor regulates the transcription of Sterol regulatory element-binding factor 1, (*Srebf1)*, which in turn, stimulates the transcription of the enzymes mentioned above to increase the lipid biosynthesis in the liver. Peroxisome proliferator-activated receptor gamma (Pparg), a master regulator of transcription, also affects the expression level of the proteins in the *de novo* lipogenesis process. In the figure the molecules studied in this research has been highlighted by bold letters in a square. The blue letters are the changes occurring in the maternal liver. In red are the changes found in fetal livers.

In order to investigate molecular signatures that may underlie the liver TG accumulation, and if the increased TG was an effect of diet or androgen injection, we analyzed the expression of the enzymes related to the *de novo lipogenesis* pathway: *Acaca, Fasn and Scd1*, and their regulators: *Lxr* and *Srebf1c*, in the liver of dams and fetuses (Fig. 6). Further, the lipid accumulation in the maternal liver does not seem to be related to an active *de novo* lipogenesis, as was demonstrated by the dysregulation of the gene expression of the enzymes that constitute the pathway. In fact, the gene expression of Acetyl-CoA Carboxylase alpha, *Acaca* was lower in DHT-injected dams fed either control chow or HF/HS. Moreover, Stearoyl-Coenzyme-A desaturase 1, *Scd1*, was also decreased in dams exposed to DHT. The regulation of the gene expression of *Acaca* and *Scd1* by androgens is supported by Kelly *et al*.^41^ who demonstrated that testosterone reversed the hepatic TG accumulation in androgen-deficient male mice^41^.

The transcription factor *Srebf1c* is recognized to be the main regulator of the transcription of the enzymes *Acaca, Fasn and Scd1*, and is at the same time regulated by *Lxr* ^42^. *Srebf1c* has been reported to have increased expression in the liver from animals exposed to a diet with a high carbohydrate content^43^. In this study, the expression level of *Srebf1c* was unaltered, probably related to the lower transcription level of *Lxr* in dams fed HF/HS. This might be a protective mechanism that reduces the *de novo* lipogenesis pathway in the liver as a response to the lipid accumulation in the tissue.

Obese dams had increased expression of *Pparg* in the liver, which is known to be related to HF/HS-induced steatosis^44, 45^. *Pparg* overexpression has been reported to result in increased expression of fat storage-inducing transmembrane protein *Fitm1* ^46^, an important precursor of lipid droplet formation^47^. Therefore, it is possible that the increased expression of *Fitm1* that we observed in the livers of dams fed with HF/HS constitutes a link between increased expression of *Pparg* and accumulation of TG in the liver of obese dams. *Apoa-1* was decreased in both HF/HS groups. ApoA1 is synthesized in liver and intestine and is an important component of HDL, a marker of cardiovascular risk^48^, suggesting a decreased capacity to remove cholesterol from macrophages in the intestine wall and its posterior excretion in the bile^49^.

Contrary to the increase in TG liver content in dams, the fetal liver TG content was decreased in dams fed with HF/HS with no significant changes in mRNA expression of genes related to *de novo lipogenesis*. The decrease in lipid accumulation contrasts what has previously been reported with higher lipid droplet accumulation in fetal livers from dams fed HF/HS^50^. Moreover, we found a negative correlation between fetal TG content and maternal pre-pregnancy weight gain (Fig. 4E) and a positive correlation during pregnancy (Fig. 4F) indicating that the TG accumulation in the fetus is determined by the weight increment of the dam before and during the pregnancy. Similar to these results, Abbott *et al*. showed a lower concentration of circulating free fatty acids (FFA) in female rhesus monkey fetuses that were prenatally androgenized (and whose mother also demonstrated an increase in their body weight) compared to the fetuses from dams injected with vehicle^24^, whereas the FFA was increased in adult female offspring^51^. Collectively, these observations raise the possibility that the lower accumulation of TG in the fetal liver in obese dams could be a result of modifications of placental transfer of FFA^50^, which is believed to be an important source for TG synthesis in the fetal liver^52^.

Another possibility is that β-oxidation is activated in the fetal liver in obese dams, which was not the focus of this study. The expression of the transcription factor *Pparg* was decreased in fetal livers exposed to DHT, which most likely is related to a direct action of the androgens on the fetal liver. An *in vitro* study demonstrated that testosterone and DHT inhibit adipocyte differentiation by decreasing the gene expression of PPARγ, C/EBPα and C/EBPβ, through the impairment of Bone Morphogenic Protein 4 (BMP4) signaling^53^. Moreover, we have demonstrated that *Pparg* mRNA expression is decreased in adipose tissue of women with PCOS compared to controls^54^. Furthermore, the fetal expression of *Srebf1c* was decreased in fetal livers from dams fed HF/HS which could be related to the lower TG content^55^, and with the absence of changes in enzymes involved in *de novo* lipogenesis in the liver^56^.

In order to investigate if HF/HS or DHT injection affect the steroidogenic environment, we assessed steroid- and glucocorticoid receptors and also enzymes related to the local steroidogenesis in the placenta. The placental protein expression of AR in DHT exposed dams was higher independent of diet, similar to what we have previously reported in the placenta from testosterone-exposed pregnant rats^22^. Of note, at adult age, female offspring displayed higher levels of red oil staining in livers and high circulating testosterone and TG in the absence of any disruption in the glucose metabolism^22^.

Because adiponectin has been proposed to be a major regulator of fetal growth and linked with maternal metabolism and placenta function^57–59^, we decided to measure circulating adiponectin in dams and adiponectin receptor 1 and 2 in the placenta. Neither circulating levels of HMW adiponectin nor protein expression of adiponectin receptor 2 in the placenta were affected by diet or the DHT injection suggesting that mothers fed HF/HS were not extremely affected in their metabolism as previously reported^58^ and may explain the lack of difference in fetal weight between the groups.

The strength of this study is the combination of two established mouse models which may reflect a pregnant woman with PCOS that frequently are overweight or obese. The dramatical increase in liver TG content in obese dams exposed to androgens is in line with the increased prevalence of nonalcoholic fatty liver disease in hyperandrogenic women with PCOS, allowing us to investigate the molecular signature and development of hepatic steatosis. The weakness is that the dose of DHT was adjusted to body weight, which could affect the variability of the results but also that we could not reproduce the LGA phenotype.

In conclusion, in our combined model, maternal obesity and androgen excess do not alter fetal and placenta weight, but negatively affect maternal and fetal liver function as demonstrated by increased TG content and dysfunctional expression of enzymes and transcription factors involved in the *de novo* lipogenesis.

## Material and Methods

### Animals

12 weeks old virgin C57/Bl6J mice were purchased from Janvier Labs (Le Genest-Saint-Isle, France). Animals were housed ten per cage under a 12 h light/dark cycle, at a temperature of 21–22 °C and 55–65% humidity. After 1 week of acclimatization, they were fed a control (CD) diet (98121701) (Research Diet, NJ, USA) containing fat: 10 kcal%, carbohydrate: 73 kcal%, and protein: 17 kcal%, or a hypercaloric (HF/HS) diet (D12079B) (Research Diet, NJ, USA) containing fat: 41 kcal%, carbohydrate: 43 kcal%, and protein; 17 kcal% together with (ii) and a 20% sucrose solution (S9378) (Sigma-Aldrich) supplemented with Vitamin mix of 10 gm/4000 kcal (V10001) (Research Diet, NJ, USA) and Mineral mix of 35 gm/4000 kcal (S10001) (Research Diet, NJ, USA). Nutrition and tap water was provided *ad libitum*. All animal procedures were approved by the Animal Ethics Committee of the University of Gothenburg (Dnr: 116–2014), in accordance with the legal requirements of the European Community (Decree 86/609/EEC).

### Experimental design and methods

Females were fed with CD (n = 25) or HF/HS (n = 27) during 4 to 10 weeks until mice in the HF/HS group had increased their weight by at least 25% compared to the beginning of the study (Supplementary Fig. 1). At this time, females in estrus phase, as determined by vaginal smears, were mated overnight with a male on the control diet. The presence of a post-copulation plug was confirmed the following morning which was defined as gestational day (GD) 0.5. Plug positive animals were placed in a separate cage and continued on the same diet. On GD 15.5 the HF/HS and CD groups were subdivided and assigned to receive a 100 µl subcutaneous injection in the interscapular area of 250 µg of 5α-Androstan-17β-ol-3-one (DHT) (cat A8380, Sigma-Aldrich, St. Louis, MO, USA), dissolved in a mixture of 5 µl Benzil Benzoate (B6630, Sigma) and 95 µl Sesame oil (S3547, Sigma) or Vehicle alone for two days (until GD 17.5). On day 18.5 animals were fasted for 4 hours and then anesthetized with 4% isoflurane inhalation. Axillary blood was collected for sex steroid analyses, and the animals were sacrificed by heart puncture. The uterus was dissected, and fetuses and placentas were collected and weighed. The fetuses were sacrificed by anesthesia inhalation prior to dissection.

### Experiment 1

#### Tissue collection

All placentas from one dam were pooled and washed in ice cold Tris-Saline buffer (10 mM Tris-HEPES, 154 mM NaCl) and homogenized on ice in buffer D (10 mM Tris-HEPES, 250 mM sucrose, 1 mM EDTA, and protease and phosphatase cocktail inhibitors (cat P8340, P5276 and P0044, Sigma) using a polytron (T 18 digital ULTRA-TURRAX®). Aliquots were snap frozen and stored at −80 °C. Fetuses were dissected from the dorsal region, and the kidneys and livers, respectively, from all pups in one litter, were pooled and snap frozen in liquid nitrogen and stored at −80 °C until further analyses. Maternal kidneys and liver were dissected, weighed, and snap frozen individually in liquid nitrogen and stored at −80 °C.

### Experiment 2

In a subset of mice under the same design (n = 5/group) lean and fat mass was examined using Dual Energy X-ray Absorption technique (DEXA) Lunar PIXImus2^tm^, (GE Medical System Lunar, Madison, USA) before the start of mating and on GD 18.5.

After 6 hours of fasting, immediately after the DEXA measurement, glucose tolerance was measured using the oral glucose tolerance test (OGTT) in conscious dams. Mice were gavaged with glucose (20% in saline), and blood glucose was measured from the tail vein at 0, 15, 30, 60, and 90 min after the glucose load. Blood glucose was measured immediately using a glucose meter (One Touch Ultra-2). At times 0 and 15 min blood was collected for insulin analysis.

In this subset of animals, the fetuses were dissected and saved in 15 ml polypropylene tubes until the next day for body composition analysis (lean and fat mass) by the Body Composition Analyzer (BCA), Bruker minispec LF110 (Bruker Biospin, Germany).

### Analytical methods

Maternal serum concentrations of progesterone, testosterone, 17β-estradiol and dihydrotestosterone were measured using a previously described validated assay at Endoceutics Inc. (Quebec City, Canada)^60^. Briefly, 75 µl of serum were treated by liquid-liquid extraction. Samples were analyzed by UPLC-ESI-MS/MS using a Shimadzu Nexera ultra high-performance liquid chromatography (UPLC) coupled with an AB Sciex Qtrap 6500™ mass spectrometer in MRM mode^60^. Chromatograms were processed and verified blindly using a validated Analyst® 1.6.2 software. Upon transfer to the validated Watson LIMS 7.4.1 data management system, data was further processed and unblinded without possible alteration.

Five µl of plasma were used for insulin measurement by the ELISA method using the Ultra-Sensitive Mouse Insulin ELISA Kit, cat# 90080 (Crystalchem.Inc, IL, USA) according to the manufacturer’s instructions. The limit of detection was 0.05 ng/ml. The intra-assay coefficient of the variations was ≤10%. The HOMA-IR index was calculated following the formula (gluc/mmol/L) x Ins(mIU/L))/22.5 assessed in mice by Bowe *et al*.^61^.

In the OGTT, the area under the curve (AUC) for glucose and insulin was calculated according to the trapezoid method:

$$A={\int }_{0}^{1.5}g(t)dt\approx \sum _{i=0}^{n}[g(i)+g(i+1)]\ast [{t}_{i+1}-{t}_{i}]/2$$

With *g(t)* the glucose concentration at time *t* and *i* the intervals of time at which measurements of glucose concentration were taken (0.0, 0.25, 0.5, 1.0 and 1.5 hours). Serum High Molecular Weight (HMW) adiponectin levels were determined using the Adiponectin (Mouse) Total, HMW ELISA kit, cat # 47-ADPMS-E01 from ALPCO (NH, USA) according to the manufacturer instructions. The intra-assay variation was <10%.

### Triglyceride liver content

Liver TGs were extracted from the dam; 100 mg of the left lateral lobe of the liver was homogenized in the Tissue Lyzer (Qiagen) for two minutes in a mixture of 5% NP-40 Alternative from Calbiochem (cat. 492016) in distilled water. Then the samples were heated at 90 °C for 5 minutes in a water bath, until the samples became cloudy, cooled down at room temperature and heated for five minutes, cooled again and centrifuged at 13,000 g × 2 minutes. The supernatant was removed and analyzed using the TG kit TR210 Randox (London, UK) according to instructions from the manufacturer.

### Western Blot

Briefly, placental homogenates were centrifuged at 13,000 g for 10 min at 4 °C, the supernatant was collected, and the protein concentration was measured using a spectrometer (Direct detect, Millipore, USA). Thirty µg of total protein was loaded in Criterion™ TGX (Tris-Glycine eXtended) Stain-Free™ precast gels (Biorad, City, Country). The gel was activated with the ChemiDoc MP imager (Biorad) and transferred to polyvinylidene difluoride membranes (PVDF) in the turbo system Trans-Blot turbo transfer (Biorad). Thereafter, membranes were blocked and incubated with primary antibodies for progesterone receptor (PR), androgen receptor (AR), estrogen receptor (ER) α and β, glucocorticoid receptor (GR), CYP11A1, 17 βHSD2 and adiponectin receptor 2 (ADIPOR2) (Supplementary Table 1). The protein expression in each sample was normalized to total amount of loaded protein in each sample using Image Lab 5.0 (Biorad).

### Periodic Acid-Schiff (PAS) staining of glomeruli

Frozen renal tissue obtained from 3 mice from each treatment group was used for immunostaining using periodic acid Schiff staining (PAS) to visualize the morphology of the glomeruli.

### RNA isolation and mRNA expression

Maternal and random pieces of the pool of fetal liver samples were homogenized in the Tissue Lyzer and using the ReliaPrep™ RNA Tissue Miniprep kit from Promega, cat #Z6112 (Madison, USA). RNA quantification was assessed by spectrophotometric measurements (Nanodrop 1000, NanoDrop Technologies). cDNA synthesis was carried out using the High Capacity RNA-to-cDNA kit, cat # 4387406 (Applied Biosystems, USA). Oligonucleotides for target genes and reference genes were purchased from Eurofins (Ebersberg, Germany) (Supplementary Table 2). Quantitative real-time PCR was performed using in Applied Biosystems® ViiA 7 RUO System (Life Technologies), and the data were analyzed by QuantStudio^TM^ Real Time PCR software version 1.2 (Life Technologies ®). We tested *Gapdh*, *Rpl0* and *Rpl19* as endogenous control, but according to NormFinder, the best one was *Rpl19* that was subsequently used as endogenous control for all our maternal and fetal samples.

### Statistical analysis

Data are presented as mean values ± SEM and were analyzed with SPSS version 21.0 (SPSS, Chicago, IL, USA). Body weight gain during pregnancy was analyzed using repeated measures ANOVA followed by Bonferroni posthoc test for group comparisons. In order to investigate if there is an interaction (simple effects) between maternal diet and maternal DHT injection or if there is a main effect of either maternal diet or maternal DHT injection, two-way ANOVA was performed followed by Bonferroni post hoc test. The results reported in brackets, [], show the degree of freedom for the groups tested, the degree of freedom of the total samples that were tested, the F value and p-value. Furthermore, if there was a significant main effect, Bonferroni posthoc test was performed. *P*-values < 0.05 were considered statistically significant. In graphs, all the data are presented as mean values ± SEM. Figures show the interaction between the diet and DHT injection effects are marked as (1) or the main effects of diet (a) or DHT treatment (b).

## Electronic supplementary material

Supplementary Information
