# Supplementary material for: The effect of androgen excess on maternal metabolism, placental function and fetal growth in obese dams
Source: Sci Rep. 2017 Aug 14;7:8066. doi: 10.1038/s41598-017-08559-w (PMC5556034; doi:10.1038/s41598-017-08559-w)

The effect of androgen excess on maternal metabolism, placental function and fetal growth in obese dams

Romina Fornes[1], Manuel Maliqueo[1,3], Min Hu[3], Laila Hadi[3], Miguel Jimenez [4], Kerstin Ebefors[3], Jenny Nyström[3], Fernand Labrie[5], Thomas Jansson[7], Anna Benrick[3,7], Elisabet Stener-Victorin[1*]

1. Department of Physiology, Department of Physiology and Pharmacology, Karolinska Institutet, 171 77 Stockholm, Sweden
2. Endocrinology and Metabolism Laboratory, Department of Medicine, West division, University of Chile, Santiago, Chile
3. Department of Physiology, Institute of Neuroscience and Physiology, Sahlgrenska Academy, University of Gothenburg, Sweden
4. Unidad Académica Multidisciplinaria Reynosa Aztlán, Universidad Autónoma de Tamaulipas, Reynosa, Tamaulipas, México
5. Laval University Research Center in Molecular Endocrinology, Oncology and Human Genomics, CHUL Research Center, Quebec, G1V 4G2, Canada.
6. Department of Obstetrics & Gynecology, Division of Reproductive Sciences, University Colorado Anschutz Medical Campus, Aurora, Colorado 80045, USA.
7. School of Health and Education, University of Skövde, 54128 Skövde, Sweden.

Corresponding author and person to whom reprint should be addressed *

Elisabet Stener-Victorin
Department of Physiology and Pharmacology
Karolinska Institutet
17177 Stockholm, Sweden
Phone: +46 705643655
E-mail: elisabet.stener-victorin@ki.se

Supplementary Tables

Antibody Table 1. Antibodies and conditions used in Western blot analysis.

Antibody Name	Company	Catalog	Dilution
Progesterone Receptor A-B	SCB	sc539	1:500
Estrogen Receptor α	SCB	sc542	1:500
Estrogen Receptor β	SCB	sc8974	1:1000
Androgen Receptor	SCB	sc-816	1:200
Glucocorticoid Receptor	SCB	sc-1004	1:500
CYP11A1	SCB	sc292456	1:500
17-β HSD type2	SCB	sc135042	1:500
Adiponectin Receptor 2	SCB	Sc514045	1:500

CYP11A1: cytochrome P450 family 11 subfamily A member 1. 17-βHSD type2: 17beta-hydroxysteroid Dehydrogenase 2.

Oligonucleotides Table 2. Oligonucleotides**.**

**Gene**			**Sequence 5´-3´**
Acetyl-CoA Carboxylase alpha	*Acaca*	Forward	GCCTCTTCCTGACAAACGAG
	Acaca	Reverse	TGACTGCCGAAACATCTCTG
Fatty acid syntase	*Fasn*	Forward	GGAGGTGGTGATAGCCGGTAT
	Fasn	Reverse	TGGGTAATCCATAGAGCCCAG
Stearoyl-CoenzimeA desaturase 1	*Scd1*	Forward	TTCTTGCGATACACTCTGGTGC
	Scd1	Reverse	CGGGATTGAATGTTCTTGTCGT
sterol regulatory element-binding factor 1	*Srebf1*	Forward	GCCCTACCGGTCTTCTATCA
	Srebf1	Reverse	TCCTGCTTGAGCTTCTGGTT
Liver X receptor (NR1H3)	*Lxr*	Forward	CTCAATGCCTGATGTTTCTCCT
	Lxr	Reverse	TCCAACCCTATCCCTAAAGCAA
Peroxisome proliferator activated receptor gamma	*Pparg*	Forward	CCCACCAACTTCGGAATCA
	Pparg	Reverse	TGCGAGTGGTCTTCCATCAC
Ribosomal protein L19	*Rpl19*	Forward	CGAAGGGTACTGCCAATGCT
	Rpl19	Reverse	TCCATGAGGATGCGCTTGTT
Apolipoprotein A-I	*Apoa1*	Forward	GGCACGTATGGCAGCAAGAT
	Apoa1	Reverse	CCAAGGAGGAGGATTCAAACTG
Fat storage-inducing transmembrane protein 1	*Fitm1*	Forward	TGCTTACGGCGCCTCTAC
	Fitm1	Reverse	CACAAACTTTATGTTGAAGAAGTTGC

Supplementary Figures

Supplementary Figure 1. Study Design. Females were fed with CD (n=25) or HF/HS (n= 27) during 4 to 10 weeks until mice in the HF/HS group gained at least 25% in weight over their own weight at the beginning of the study. In a first experiment, the obese animal together with a CD fed mouse were subjected to a DEXA and mated overnight with a male on control diet. The presence of a post copulation plug was confirmed the following morning which was defined as gestational day (GD) 0.5. During pregnancy At GD 15.5 the HF/HS and CD groups were subdivided and assigned to receive a 100 µl subcutaneous injection in the interscapular area of 250 µg/kg of 5α-Androstan-17β-ol-3-one (DHT) or Vehicle alone for two days (until GD 17.5). On day 18.5 animals were fasted for 4 hours, a second DEXA and OGTT was done. After that the pregnant female were anesthetized with 4% isoflurane inhalation, axillar blood was collected for sex steroid analyses and finally sacrificed by heart puncture. The uterus was dissected and fetuses and placentas were collected and weighed. Further, the fetuses in this first experiment were assessed by Body composition analyses. Following the same design, a second experiment was done where the maternal liver and kidneys were removed from dams, and placenta and fetal liver were collected to perform qPCR and Western blot. All the fetuses were sacrificed by anesthesia inhalation immediately after collection.

Supplementary Figure 2. CYP11A and 17-HSD II in placenta homogenates at GD 18.5 (A) Cholesterol side-chain cleavage enzyme (CYP11A). (B) 17 beta-Hydroxysteroid dehydrogenase type 2 (17βHSD2) in placenta homogenates from pregnant mice on GD 18.5 (CD-Veh, n=8; HF/HS, n=8; CD-DHT, n=8; HF/HS-DHT, n=10). Protein expression was quantified by western blot and normalized by total protein loaded in each line in the stain free blot. Values are means ± SEM.

Supplementary Figure 3. Adiponectin metabolism at day GD 18.5. (A)High Molecular Adiponectin in maternal serum and (B) protein expression of Adiponectin receptor 2 in placenta homogenates on GD 18.5. (CD-Veh, n=8; HF/HS, n=8; CD-DHT, n=8; HF/HS-DHT, n=10). Protein expression was quantified by western blot and normalized by total protein loaded in each line in the stain free blot. Every blot image was cropped for publication and the full image of every blot is showed in Supplementary Figure 5. Values are means ± SEM.

Supplementary Figure 4. Representative western blot for (A). Androgen receptor (AR), (B) Progesterone receptor A (PRA), (C) Progesterone receptor B (PRB), (D) Estrogen receptor α (ERα), (E) Estrogen receptor β (ERβ), (F) Glucocorticoids receptor (GR), (G) Adiponectin Receptor 2 (AdipoR2), (H) Cholesterol side-chain cleavage enzyme (CYP11A1), (I) 17 beta-Hydroxysteroid dehydrogenase type 2 (17βHSD2). The blots were cropped at about 75 kD and probed with the respective antibody

according to the molecular weight. Images for progesterone receptor were captured at different times (40, 60 and 120 sec) in order to get the better resolution for the PRB. Images from 120 sec were selected for analysis.

Supplementary Figure 1

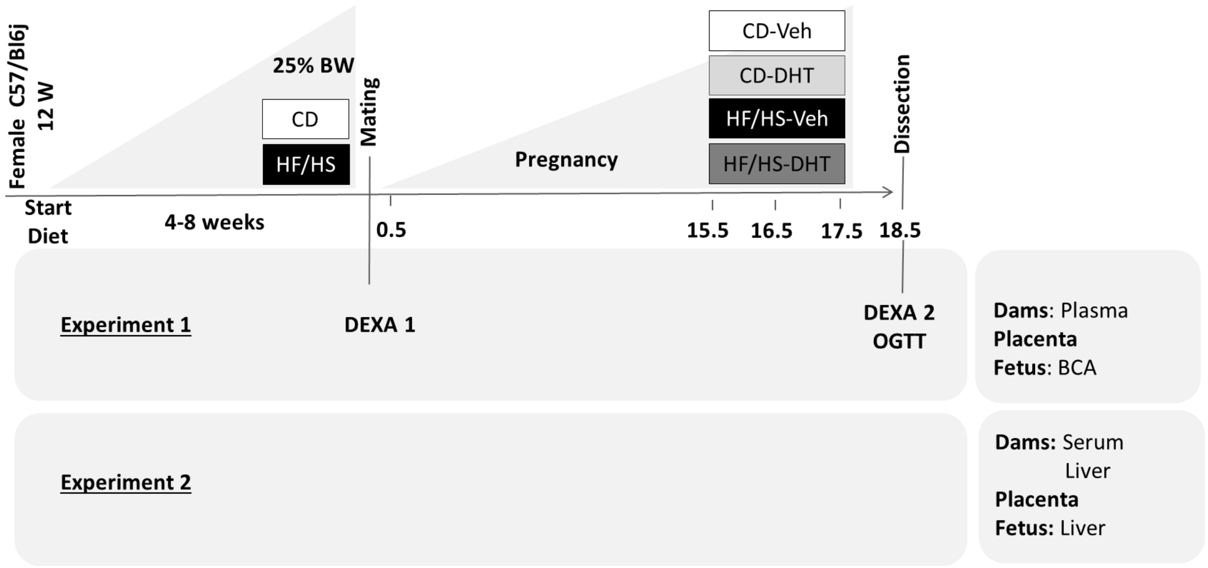

Supplementary Figure 2

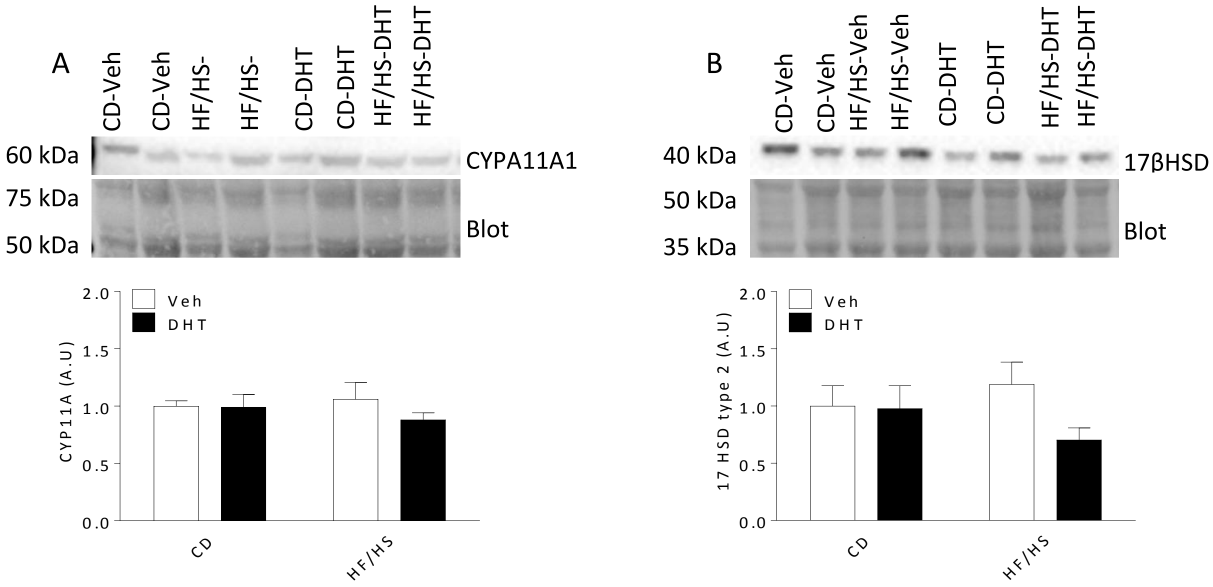

Supplementary Figure 3

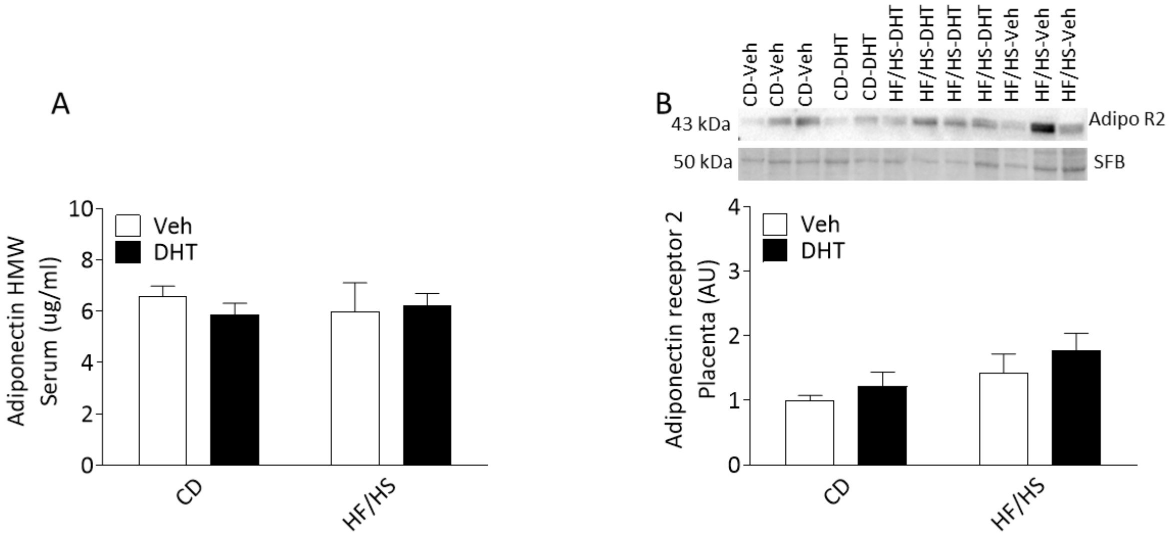

Supplementary Figure 4

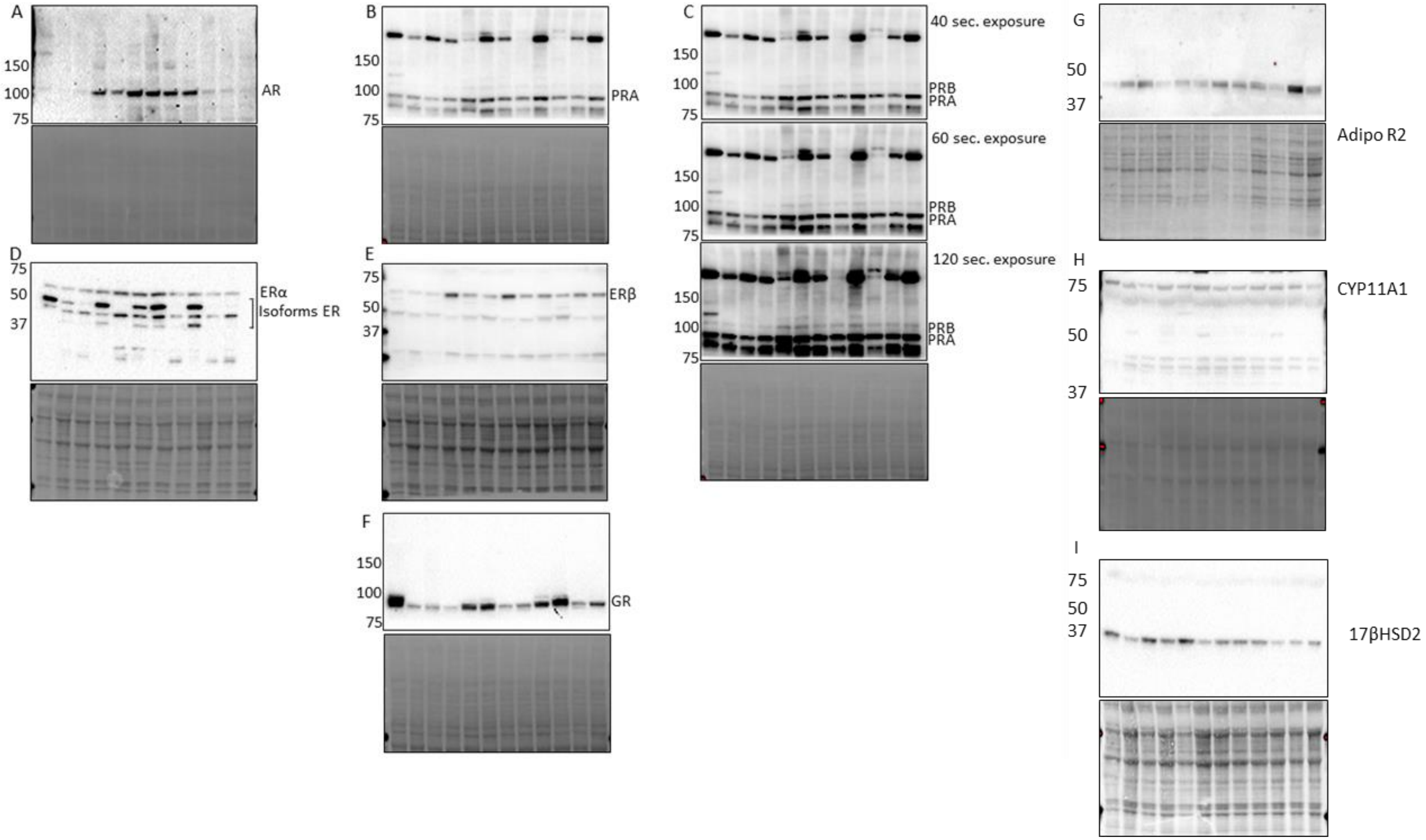

Supplement: Supplementary file 1 — Supplementary Information [file 41598_2017_8559_MOESM1_ESM.pdf]
